# Risk factor analysis for stunting incidence using sparse categorical principal component logistic regression

**DOI:** 10.1016/j.mex.2025.103186

**Published:** 2025-01-27

**Authors:** Anna Islamiyati, Muhammad Nur, Abdul Salam, Wan Zuki Azman Wan Muhamad, Dwi Auliyah

**Affiliations:** a Department of Statistics, Faculty of Mathematical and Natural Sciences, Hasanuddin University, Makassar 90245, Indonesia; b Department of Mathematics, Faculty of Mathematical and Natural Sciences, Hasanuddin University, Makassar 90245, Indonesia; c Department of Nutrition, Faculty of Public Health, Hasanuddin University, Makassar 90245, Indonesia; d Institute of Engineering Mathematics, Universiti Malaysia Perlis, Arau 02600, Malaysia

**Keywords:** Binary logistic, Categorical predictor, Multicollinearity, Sparse Categorical PCA, Sparse Categorical Principal Component Logistic Regression

## Abstract

The risk factors for stunting incidence involve categorical data in both the response and predictor variables. Therefore, we developed a sparse categorical principal component logistic regression model capable of handling data with multicollinearity. The parameters of the sparse categorical principal component logistic regression model were estimated using the maximum likelihood method and the Newton-Raphson iterative approach. The analysis yielded a likelihood ratio value of 144.81 and a chi-square statistic value of 11.07, indicating that all factors included in the model are statistically significant. The results highlight that medical history, inadequate complementary feeding, formula feeding, lack of complementary feeding programs, and lack of iron supplementation for mothers are highly associated with the risk of stunting in toddlers. This emphasizes the need for attention to maternal nutrition from pregnancy through breastfeeding, as well as the nutrition of the toddler. Some important points proposed in this method are:

• Stunting data consists of categorical variables containing multicollinearity.
• The method applied is sparse logistic regression combined with categorical principal component analysis.
• Analysis of risk factors for stunting in toddlers is based on the child's own condition, as well as parental factors, namely age, education, and intake of additional food and supplementary tablets during pregnancy.

## Graphical abstract

**Figure:**
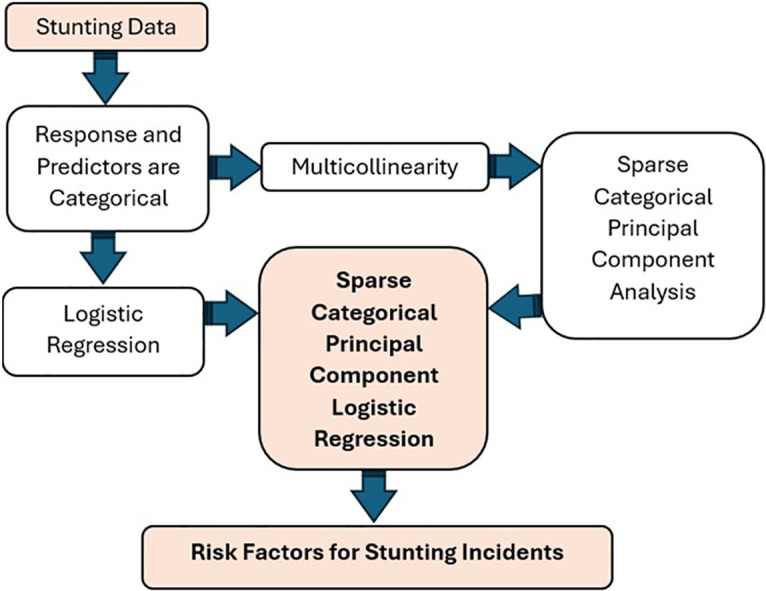

Specifications table

**Table:** 

Subject area:	Mathematics and Statistics
More specific subject area:	Statistics modelling
Name of your method:	Sparse Categorical Principal Component Logistic Regression
Name and reference of original method:	None
Resource availability:	Software R

## Background

Stunting is a chronic growth disorder in children caused by prolonged malnutrition and frequent infections, particularly during the critical 1000 days of life. This condition is characterized by a height below the age-standard reference, reflecting the accumulated negative effects of nutrient deficiencies and repeated infections, such as diarrhea and respiratory tract infections, which can hinder nutrient absorption [[1], [2]]. Limited access to economic resources, low maternal education levels, and poor access to quality healthcare services further exacerbate the risk of stunting in children [3]. In addition to internal factors, environmental conditions such as clean water and adequate sanitation are crucial, as unhealthy environments can serve as sources of infections that impair child development [4]. Various studies emphasize that improving maternal education and providing adequate nutritional interventions during pregnancy and childhood can significantly reduce stunting rates, with environmental improvements being a key preventive step [[5], [6]].

Many researchers have studied stunting data, for example, a study of variations in weight and height growth in children in rural South Africa using the SITAR model [6]. Analysis of stunting by age in the South Asian Region [7] and detection of child growth retardation in Indonesia using penalized spline regression [8]. However, stunting data often involves categorical data on response and predictor variables, requiring a more precise statistical approach to analyzing it. Logistic regression is a commonly used statistical method to predict categorical response variables based on one or more predictor variables. Specifically, binary logistic regression is frequently used to analyze the relationship between predictors and a dichotomous response or a categorical variable with two levels [[9], [10]]. In contrast, ordinal logistic regression is used for data with ordered categories [11]. One of the critical assumptions in logistic regression is the absence of multicollinearity among predictors, as multicollinearity can distort the accuracy of the regression coefficients, leading to incorrect interpretations, particularly with odds ratios [12]. This issue frequently arises in high-dimensional datasets, where strong correlations between predictors are challenging to avoid and can affect the stability of the model [13]. Principal component analysis has long been used as a dimensionality reduction technique to address multicollinearity, transforming the predictor variables into uncorrelated principal components [14]. With principal component analysis, high multicollinearity data can be converted into principal components free of correlation and subsequently used as predictors in logistic regression.

In high-dimensional datasets, such as those used in genomics or pattern recognition applications, multicollinearity can significantly impact the interpretation and stability of the model. Classic principal component analysis has limitations, as the principal components produced are linear combinations of all variables, making the component loadings difficult to interpret [15]. One approach to address this issue is sparse principal component analysis, which introduces a sparsity penalty to reduce dimensionality and select relevant features for logistic regression models. Sparse principal component analysis offers an alternative by combining dimensionality reduction and sparsity, ensuring that only significant variables are included in each principal component while diminishing the weights of less relevant variables to zero [16]. Sparse principal component analysis has proven to be more effective for high-dimensional data modeling, as it produces principal components that are more interpretable for categorical responses, facilitating better insights into the underlying patterns in the data [17].

As the need for models capable of handling categorical predictors increases, such as in the case of risk factors for stunting, one promising approach is categorical principal component analysis, introduced by Kemalbay and Korkmazoglu [18]. Given that sparse principal component analysis can enhance the interpretability and variable selection in binary logistic regression models [19], developing a sparse principal component analysis logistic regression model for categorical predictors offers a valuable solution for analyzing risk factors for stunting. This method leverages the sparsity constraint to select significant variables while reducing dimensionality, thus improving the stability and interpretability of the model when applied to high-dimensional categorical data. This approach could provide more accurate insights into the factors contributing to stunting by focusing on the most relevant predictors.

## Method details

### Categorical principal component analysis

One of the violations of assumptions that often occurs in data is the occurrence of multicollinearity, namely a high correlation between predictors. In such cases, principal component analysis is an old statistical technique that can be used by linearly transforming the original set of variables into a smaller set of uncorrelated variables, which can still represent the information of the original set of variables. These new variables are called principal components. Let R represent the correlation matrix of predictor variables $X=[X_{1},X_{2},\ldots ,X_{p}]$, and the pairs of eigenvalues and eigenvectors are $\left(\lambda_{1},v_{1}\right),\left(\lambda_{2},v_{2}\right),\ldots ,\left(\lambda_{p},v_{p}\right)$ where p is the number of predictor variables and $\lambda_{1}\geq \lambda_{2}\geq \ldots \;\geq \lambda_{p}$. The following Eq. (1) can define the principal components formed as linear combinations.

(1) $$Z_{r}={v^{\prime }}_{r}\mathrm{rX}=v_{r1}X_{1}+v_{r2}X_{2}+\ldots +v_{rp}X_{p},\;r=1,2,\ldots ,\;p$$

Eq. (1) can be expressed in matrix form as in Eq. (2).

(2) $${v^{\prime }}_{r}X=\left[\begin{array}{cccc} v_{11} & v_{21} & \ldots & v_{p1}\\ v_{12} & v_{22} & \ldots & v_{p2}\\ \vdots & \vdots & \ddots & \vdots \\ v_{1p} & v_{2p} & \ldots & v_{pp} \end{array}\right]\left[\begin{array}{c} X_{1}\\ X_{2}\\ \begin{array}{c} \vdots \\ X_{p} \end{array} \end{array}\right]$$

where $Z_{r}$ represents the r-th principal component, *r* is the index for the principal components from 1 to *p* with r≤p. The criterion for selecting principal components is based on the cumulative proportion of the total variance that can be explained by the selected components, with a minimum threshold of 80% explained variance [20].

Next, categorical principal component analysis was developed, a method for dealing with multicollinearity in categorical data by applying optimal scaling, which converts categorical labels into numeric values while maximizing the variance between variables [21]. There are *n* individuals with *p* predictor variables, where each variable is defined by $X_{j}$ for j=1,2,…,p. If $X_{j}$ is a nominal or ordinal scale, then the optimal linear transformation is observed for each score by converting them into categorical quantifications. The variable $X_{j}$ resulting from quantification is denoted by $X^{*}$.

Let $C_{j}$ be a matrix of size $v_{j\;}x\;1$, where $v_{j\;}$ is the number of categories for each variable $X_{j}$, and the values of $C_{j\;}$ are consecutive integers. To find the solution for $X_{j}$ and the values of $C_{j}$, the problem can be formulated by minimizing the function in Eq. (3) through the alternating least square algorithm. It includes the least squares approximation of each parameter by updating each parameter matrix [22].

(3) $$\sigma \left(\bar{X};C_{j}\right)=\frac{1}{n}\sum_{j=1}^{p}\frac{1}{j}\left\{tr{\left(\bar{X}-G_{j}C_{j}\right)}^{T}\left(\bar{X}-G_{j}C_{j}\right)\right\}$$

where $G_{j}$ is the indicator matrix of $X_{j}$ which has a value of 1 if the value of $X_{j}$ is included in the category $c_{j}$, and a value of 0 if the value of $X_{j}$ is not included in the category $c_{j}.$ The optimal value of $C_{j}$ resulting from quantification can be denoted by $C_{j}^{*}$. Next, the optimal quantification process is obtained through Eq. (4), namely:

(4) $${\left(G_{j}^{T}G_{j}\right)}^{-1}G_{j}^{T}{\bar{X}}^{\left(t-1\right)}\;=C_{j}^{\left(t\right)}$$

Where *t* is the iteration. The value of $C_{j}^{\left(t\right)}$ at t=1 is obtained based on ${\bar{X}}^{\left(0\right)}$. The variable ${\bar{X}}^{\left(0\right)}$ is the average of $X_{j}^{\left(0\right)},\;j=1,2,\ldots ,p$. After that, we determine the value of $X_{j}^{\left(t\right)}$ based on Eq. (5).

(5) $$X_{j}^{\left(t\right)}=G_{j}C_{j}^{\left(t\right)}$$

The iteration process is stopped when $\sigma \left({\bar{X}}^{\left(t-2\right)};C_{j}^{\left(t-1\right)}\right)-\sigma \left({\bar{X}}^{\left(t-1\right)};C_{j}^{\left(t\right)}\right)<\epsilon$, $\epsilon =10^{-6}$. The value of $C_{j}^{*}$ and $X^{*}$ are obtained when the iteration process is stopped.

In categorical principal component analysis, the correlation matrix R is computed among the quantified variables $X^{*}$ as in Eq. (6).

(6) $$R=\frac{1}{n-1}{X^{*}}^{T}X^{*}$$

with $\lambda_{j\;}=\;\lambda_{1},\;\lambda_{2},\ldots ,\;\lambda_{p}$ as the eigenvalues of the correlation matrix R and $a_{j}=\;{\left(a_{1},\;a_{2},\ldots ,\;a_{p}\right)}^{T}$ as the eigenvector corresponding to the eigenvalue $\lambda_{j\;}$. If the eigenvalues $\lambda_{j\;}$ are ordered from the largest to the smallest, then the ordering of the principal components corresponds to $\lambda_{j\;}$. This study uses a minimum cumulative variance proportion of 80% for forming categorical principal components. If the principal components for the quantified predictor variables are expressed as $Z_{k}$ formed are k=1,2,…,r, then $Z_{k}$ can be expressed by the following Eq. (7).

(7) $$Z_{k}={a^{\prime }}_{k}X^{*}=a_{k\left(1\right)}X_{1}^{*}+a_{k\left(2\right)}X_{2}^{*}+\ldots +a_{k\left(p\right)}X_{p}^{*},\;k=1,\;2,\;\ldots ,r$$

### Sparse principal component analysis

Sparse principal component analysis is an extension of the principal component analysis method that can eliminate ineffective variables from the principal components by reducing the loading values to zero [16]. The sparse principal component analysis uses the elastic net, developed from the least absolute shrinkage and selection operator method, to produce modified principal components from sparse loading values by combining $L_{1}$*-*norm and squared $L_{2}$*-*norm constraints on the elastic net estimator δ [23]. The $L_{1}$*-*Norm constraint can result in a simpler model by shrinking some δ values to exactly zero, while the squared $L_{2}$*-*Norm constraint generates a model that does not select variables but enhances the grouping and shrinking effects of δ [24].

The sparse principal component analysis algorithm for reducing data dimensionality using the naive elastic net estimator is formulated with the following steps [25]:

1. Let $A=[A_{1},\ldots ,\;A_{r}]$, representing the loading values of each principal component.
2. The naive elastic net estimator for k=1,2,…,r is calculated by the following Eq. (8).

(8) $$\hat{\delta_{k}}=\underset{\delta_{k}}{\text{arg}\;\text{min}}\left\{{\left(A_{k}-\delta_{k}\right)}^{T}{X^{*}}^{T}X^{*}\left(A_{k}-\delta_{k}\right)+l_{2}∥\delta_{k}{∥}_{2}^{2}+l_{1}∥\delta_{k}{∥}_{1}\right\}$$

where $∥\delta_{k}{∥}_{2}^{2}=\sum_{j=1}^{p}\delta_{jk}^{2}$, $∥\delta_{k}{∥}_{1}=\sum_{j=1}^{p}\left|\delta_{jk}\right|$, $l_{1}$ and $l_{2}$ are positive numbers, tuning parameters determined by considering the parsimony principle.

3. For each δ obtained from step 2, calculate the SVD of ${X^{*}}^{T}X^{*}B$ with ${X^{*}}^{T}X^{*}B=\mathrm{UD}V^{T}$, to obtain $A=UV^{T}$.
4. Repeat steps 2–3 until δ converges.
5. Perform normalization: ${\hat{v}}_{k}=\frac{\delta_{k}}{∥\delta_{k}∥};k=1,\ldots ,r$.

Next, the optimal sparse principal component $Z_{k}^{*}\;$can be obtained based on the values of ${\hat{v}}_{k}$ which is the result of normalizing the values of $\delta_{k}\;$by choosing the values of $l_{1}$ and $l_{2}$ to obtain a simple model with a minimum cumulative variance proportion of 80%. The sparse categorical principal component is shown in Eq. (9).

(9) $$Z_{k}^{*}={\hat{v}}_{k}X^{*}={\hat{v}}_{k\left(1\right)}X_{1}^{*}+{\hat{v}}_{k\left(2\right)}X_{2}^{*}+\ldots +{\hat{v}}_{k\left(p\right)}X_{p}^{*}$$

### Principal component logistic analysis

Principal component logistic regression aims to improve parameter estimation in logistic regression models with multicollinearity among predictor variables by using the principal components of these predictors. The equation for the principal component logistic regression model is shown in Eq. (10) [25].

(10) $$\pi \left(Z\right)=\frac{\text{exp}\{\beta_{0}+\sum_{k=1}^{r}\sum_{j=1}^{p}Z_{k}v_{jk}\beta_{j}\}}{1+\text{exp}\{\beta_{0}+\sum_{k=1}^{r}\sum_{j=1}^{p}Z_{k}v_{jk}\beta_{j}\}}=\frac{\text{exp}\{\beta_{0}+\sum_{k=1}^{r}Z_{k}\gamma_{k}\}}{1+\text{exp}\{\beta_{0}+\sum_{k=1}^{r}Z_{k}\gamma_{k}\}}$$

where π(Z) is the probability of success and $\beta_{0},\;\gamma_{1},\;\ldots ,\;\gamma_{r}$ are the regression parameters. The principal component logistic regression model can be expressed in terms of the linear relationship between log odds and predictor variables through the logit transformation $g=\ln(\frac{\pi (Z)}{1-\pi (Z)})$. The model is shown in Eq. (11).

(11) $$g=\beta_{0}+\sum_{k=1}^{r}Z_{k}\gamma_{k}+\varepsilon$$

where g is the probability of a successful outcome when Y=1, and $\gamma_{k}$ is the logistic regression coefficient based on the principal components formed.

### Parameter estimation of the sparse categorical principal component logistic regression model

The model obtained for sparse categorical principal component logistic regression analysis is shown in Eq. (12).

(12) $$g=\beta_{0}+\sum_{k=1}^{r}Z_{k}^{*}\gamma_{k}+\varepsilon$$

where g represents the probability of success for Y=1, $\beta_{0}$ is the intercept or the value of g when all $Z_{k}^{*}$ are zero, $Z_{k}^{*}$ is the optimal sparse principal component, and $\gamma_{k}$ is the regression coefficient that measures the effect of $Z_{k}^{*}$ on the probability of g. The parameters ($\beta_{0},\gamma_{1},\ldots ,\gamma_{r})$ are estimated using the maximum likelihood estimation method by maximizing the likelihood function in Eq. (13).

(13) $$f\left(\left(\beta_{0},\gamma_{1},\gamma_{2},\ldots ,\gamma_{r}\right);Y_{i}\right)=\prod_{i=1}^{n}f\left(Y_{i}\right)=\prod_{i=1}^{n}{\left(\frac{\pi \left(Z_{i}^{*}\right)}{1-\pi \left(Z_{i}^{*}\right)}\right)}^{Y_{i}}\left(1-\pi \left(Z_{i}^{*}\right)\right)$$

Therefore, we obtain the log-likelihood function as in Eq. (14).

(14) $$\begin{array}{c} \ln L\left(\left(\beta_{0},\gamma_{1},\gamma_{2},\ldots ,\gamma_{r}\right);Y_{i}\right)=ln\prod_{i=1}^{n}{\left(\frac{\pi \left(Z_{i}^{*}\right)}{1-\pi \left(Z_{i}^{*}\right)}\right)}^{Y_{i}}\left(\frac{\pi \left(Z_{i}^{*}\right)}{1-\pi \left(Z_{i}^{*}\right)}\right))\\ =\sum_{i=1}^{n}\left[Y_{i}\left(\beta_{0}+\sum_{k=1}^{r}Z_{k}^{*}\gamma_{k}\right)-ln\left(1+exp\left(\beta_{0}+\sum_{k=1}^{r}Z_{k}^{*}\gamma_{k}\right)\right)\right] \end{array}$$

Next, Eq. (14) is differentiated for its parameters, shown in (15), (16), (17).

(15) $$\frac{\partial ln\left(\left(\beta_{0},\gamma_{1},\gamma_{2},\ldots ,\gamma_{r}\right);Y_{i}\right)}{\partial \beta_{0}}=\sum_{i=1}^{n}\left[Y_{i}-\frac{exp\left(\beta_{0}+\sum_{k=1}^{r}Z_{k}^{*}\gamma_{k}\right)}{1+exp\left(\beta_{0}+\sum_{k=1}^{r}Z_{k}^{*}\gamma_{k}\right)}\right]$$

(16) $$\frac{\partial ln\left(\left(\beta_{0},\gamma_{1},\gamma_{2},\ldots ,\gamma_{r}\right);Y_{i}\right)}{\partial \gamma_{1}}=\sum_{i=1}^{n}\left[Y_{i}Z_{i1}^{*}-Z_{i1}^{*}\frac{exp\left(\beta_{0}+\sum_{k=1}^{r}Z_{k}^{*}\gamma_{k}\right)}{1+exp\left(\beta_{0}+\sum_{k=1}^{r}Z_{k}^{*}\gamma_{k}\right)}\right]$$

(17) $$\frac{\partial ln\left(\left(\beta_{0},\gamma_{1},\gamma_{2},\ldots ,\gamma_{r}\right);Y_{i}\right)}{\partial \gamma_{r}}=\sum_{i=1}^{n}\left[Y_{i}Z_{ir}^{*}-Z_{ir}^{*}\frac{exp\left(\beta_{0}+\sum_{k=1}^{r}Z_{k}^{*}\gamma_{k}\right)}{1+exp\left(\beta_{0}+\sum_{k=1}^{r}Z_{k}^{*}\gamma_{k}\right)}\right]$$

Since the first derivatives of the log-likelihood function do not provide a solution, the Newton-Raphson iteration is used for parameter estimation ($\beta_{0},\gamma_{1},\ldots ,\gamma_{r})$. The Newton-Raphson method requires both the first and second derivatives of the log-likelihood function. The second derivative of the log-likelihood function for ($\beta_{0},\gamma_{1},\ldots ,\gamma_{r})$ can be seen in (18), (19), (20).

(18) $$\frac{\partial^{2}\text{ln}L\left(\left(\beta_{0},\gamma_{1},\ldots ,\gamma_{r}\right);Y_{i}\right)}{\partial^{2}\beta_{0}}=-\sum_{i=1}^{n}\left[\frac{exp\left(\beta_{0}+\sum_{k=1}^{r}Z_{k}^{*}\gamma_{k}\right)}{1+exp\left(\beta_{0}+\sum_{k=1}^{r}Z_{k}^{*}\gamma_{k}\right)}\right]$$

(19) $$\frac{\partial^{2}\text{ln}L\left(\left(\beta_{0},\gamma_{1},\ldots ,\gamma_{r}\right);Y_{i}\right)}{\partial \beta_{0}\partial \gamma_{l}}=-\sum_{i=1}^{n}\left[Z_{il}^{*}\frac{exp\left(\beta_{0}+\sum_{k=1}^{r}Z_{k}^{*}\gamma_{k}\right)}{{\left(1+exp\left(\beta_{0}+\sum_{k=1}^{r}Z_{k}^{*}\gamma_{k}\right)\right)}^{2}}\right]$$

(20) $$\frac{\partial^{2}\text{ln}L\left(\left(\beta_{0},\gamma_{1},\ldots ,\gamma_{r}\right);Y_{i}\right)}{\partial \gamma_{l}\partial \gamma_{k}}=-\sum_{i=1}^{n}\left[Z_{il}^{*}Z_{ik}^{*}\frac{exp\left(\beta_{0}+\sum_{k=1}^{r}Z_{k}^{*}\gamma_{k}\right)}{{\left(1+exp\left(\beta_{0}+\sum_{k=1}^{r}Z_{k}^{*}\gamma_{k}\right)\right)}^{2}}\right]$$

for l,k=1,2,…,r.

After obtaining the first and second derivatives of the log-likelihood function concerning ($\beta_{0},\gamma_{1},\ldots ,\gamma_{r})$, the parameter estimates (${\hat{\beta }}_{0},{\hat{\gamma }}_{1},\ldots ,{\hat{\gamma }}_{r})$ are generally obtained using the Newton-Raphson iteration as in Eq. (21).

(21) $$\left[\begin{array}{c} {\hat{\beta }}_{0\left(t+1\right)}\\ {\hat{\gamma }}_{1\left(t+1\right)}\\ \begin{array}{c} \vdots \\ {\hat{\gamma }}_{r\left(t+1\right)} \end{array} \end{array}\right]=\left[\begin{array}{c} {\hat{\beta }}_{0\left(t\right)}\\ {\hat{\gamma }}_{1\left(t\right)}\\ \begin{array}{c} \vdots \\ {\hat{\gamma }}_{r\left(t\right)} \end{array} \end{array}\right]-{D_{2}}^{-1}D_{1}$$

Where $D_{2}$ is the matrix of second derivatives of the log-likelihood function concerning ($\beta_{0},\gamma_{1},\ldots ,\gamma_{r})$, and $D_{1}$ is the matrix of first derivatives of the log-likelihood function concerning ($\beta_{0},\gamma_{1},\ldots ,\gamma_{r})$.

The estimated model for sparse categorical principal component logistic regression analysis can be seen in Eq. (22).

(22) $$g={\hat{\beta }}_{0}+Z_{1}^{*}{\hat{\gamma }}_{1}+Z_{2}^{*}{\hat{\gamma }}_{2}+\ldots +Z_{3}^{*}{\hat{\gamma }}_{3}$$

In Eq. (22), the regressed variables are the optimal sparse principal component variables formed from the sparse categorical principal component analysis. Therefore, Eq. (22) needs to be transformed back into the original X variables so that the response variable Y can be clearly expressed in terms of the predictor variable X.

## Method validation

### Data description

This study uses data on stunting occurrences in Maginti District in 2023 from the West Muna District Health Office, Southeast Sulawesi Province, Indonesia. The categorical predictor variables in this study are risk factors that may lead to stunting. The outcome variable for stunting consists of 0 (non-stunted) and 1 (stunted). The predictor variables include gender, birth height, birth weight, history of illness, age at the introduction of solid food, formula milk consumption, food during pregnancy, mother received iron tablets, father and mother's age, father and mother's highest education level, and the drinking water consumed. This study uses R software in analyzing data.

Based on the data, the frequency distribution of stunting occurrences among the respondents is shown in Table 1. There were 296 respondents; 84.8% (251) were not identified as stunted, while 15.2% (45) were identified as stunted.

**Table 1:** Frequency distribution of stunting occurrence.

Stunting Status	Frequency	Percentage (%)
Non-stunted	251	84.8
Stunted	45	15.2
Total	296	100

Table 2 shows that from 15.2% of respondents who were indicated as stunted, the incidence in girls and boys is the same, namely 7.8% and 7.4%. Furthermore, the high percentage of stunting in children who have low height and birth weight is 14.5% and 12.8%, respectively. Furthermore, 12.2% of children have a history of illness, 11.8% receive additional food that is not according to their age, and 11.5% consume formula milk. For the child's mother, 7.8% do not receive additional food, and 8.8% do not receive iron tablets. Furthermore, for the age of the parents, the highest percentage of children indicated as stunting have fathers who are over 40 years old, namely 6.1%. As for the mother's age, the highest is between the ages of 21 – 35 years, namely around 7.1%. For the last education of the parents, the highest percentage occurs in children with father and mother education at the high school level, namely 6.7% and 6.4%, respectively.

**Table 2:** Frequency distribution of each predictor variable based on stunting incidence.

Variable	Category	Stunting Status	
		Non-stunted	Stunted
Gender (*X*_1_)	Female Male	50.3% 34.5%	7.8% 7.4%
Birth Height (*X*_2_)	Abnormal (Male <48 cm, Female <47.3 cm) Normal (Male ≥48 cm, Female ≥47.3 cm)	17.2% 67.6%	14.5% 0.7%
Birth Weight (*X*_3_)	Abnormal (<2.5 kg) Normal (≥2.5 kg)	19.3% 65.5%	12.8% 2.4%
History of Illness (*X*_4_)	No Yes	52.7% 32.1%	3% 12.2%
Age of Introduction to Solid Foods (*X*_5_)	Appropriate (6 months) Inappropriate (<6 months & >6 months)	47% 37.8%	3.4% 11.8%
Formula Milk Consumption (*X*_6_)	Yes No	65.5% 19.3%	11.5% 3.7%
Mother Received Additional Food During Pregnancy (*X*_7_)	Yes No	57.4% 27.4%	7.4% 7.8%
Mother Received Iron Tablets (*X*_8_)	Yes No	57.4% 27.4%	6.4% 8.8%
Father's Age (*X*_9_)	<25 years 25 – 40 years >40 years	6.7% 48.6% 29.4%	5.1% 4.1% 6.1%
Mother's Age (*X*_10_)	<21 years 21 – 35 years >35 years	7.4% 59.5% 17.9%	6.4% 7.1% 1.7%
Father's Highest Education (*X*_11_)	Elementary School Middle School High School University	4.1% 7.4% 47.6% 25.7%	1.4% 5.4% 6.7% 1.7%
Mother's Last Education Level (*X*_12_)	Elementary School Middle School High School University	3% 8.8% 51.7% 21.3%	1.7% 6.1% 6.4% 1%
Type of Drinking Water Used (*X*_13_)	Packaged Gallon Refillable Gallon Boiled Water	3.4% 70.6% 10.8%	0% 5.4% 9.8%

### Risk factor analysis for stunting occurrence

In the data analysis process, the use of categorical principal component analysis begins with the formation of optimal quantification of each category of predictor variables. It is known that $X^{*}$ is the variable of the optimal quantification result obtained based on the value of $C_{j}^{*}$ from the iteration process as in Eq. (5). The values for each variable $X^{*}$ are shown in Table 3.

**Table 3:** Optimal category quantification $C_{j}^{*}$ for each category in $X^{*}.$

Variable	Category	Optimal Quantification
Gender (*X*_1_)	Female Male	−0.049 0.068
Birth Height (*X*_2_)	Abnormal (Male <48 cm, Female <47.3 cm) Normal (Male ≥48 cm, Female ≥47.3 cm)	−0.085 0.039
Birth Weight (*X*_3_)	Abnormal (<2.5 kg) Normal (≥2.5 kg)	−0.084 0.039
History of Illness (*X*_4_)	No Yes	−0.051 0.065
Age of Introduction to Solid Foods (*X*_5_)	Appropriate (6 months) Inappropriate (<6 months & >6 months)	−0.057 0.058
Formula Milk Consumption (*X*_6_)	Yes No	−0.031 0.106
Mother Received Additional Food During Pregnancy (*X*_7_)	Yes No	−0.042 0.079
Mother Received Iron Tablets (*X*_8_)	Yes No	−0.043 0.077
Father's Age (*X*_9_)	<25 years 25 – 40 years >40 years	−0.158 0.021 0.021
Mother's Age (*X*_10_)	<21 years 21 – 35 years >35 years	−0.145 0.002 0.029
Father's Highest Education (*X*_11_)	Elementary School Middle School High School University	−0.157 −0.102 0.018 0.042
Mother's Last Education Level (*X*_12_)	Elementary School Middle School High School University	−0.145 −0.106 0.023 0.041
Type of Drinking Water Used (*X*_13_)	Packaged Gallon Refillable Gallon Boiled Water	−0.048 −0.028 0.113

Next, through categorical principal component analysis, five optimal principal components, $Z_{k}$, k=1,2,…,5, were obtained, which collectively explain a cumulative variance percentage of 85.44%. The loading values $A_{\mathrm{jk}}$, which represent the correlation between $Z_{k}$ and $X_{j}^{*}$, are shown in Table 4.

**Table 4:** Optimal loading value.

**Loading value**	${Z_{1}}^{*}$	${Z_{2}}^{*}$	${Z_{3}}^{*}$	${Z_{4}}^{*}$	${Z_{5}}^{*}$
${X_{1}}^{*}$	0.488	0.208	0.495	0.338	−0.407
${X_{2}}^{*}$	−0.716	−0.321	0.333	−0.215	−0.358
${X_{3}}^{*}$	−0.741	−0.365	0.281	−0.199	−0.320
${X_{4}}^{*}$	0.729	0.412	0.276	0.280	−0.028
${X_{5}}^{*}$	0.694	0.414	0.238	0.169	0.039
${X_{6}}^{*}$	0.629	0.384	−0.004	−0.405	−0.231
${X_{7}}^{*}$	0.795	0.241	−0.212	−0.374	−0.175
${X_{8}}^{*}$	0.776	0.125	−0.198	−0.334	−0.152
${X_{9}}^{*}$	−0.528	0.619	0.325	−0.234	0.315
${X_{10}}^{*}$	−0.515	0.623	0.341	−0.312	0.207
${X_{11}}^{*}$	−0.632	0.530	−0.298	0.202	−0.135
${X_{12}}^{*}$	−0.664	0.621	−0.191	0.156	−0.157
${X_{13}}^{*}$	0.621	−0.526	0.315	−0.048	0.298

Table 4 needs to be simplified using sparse principal component analysis so that each $Z_{k}$ consists only of a few $X_{j}^{*}$ variables that contribute significantly to the optimal principal component $Z_{k}$. The initial analysis steps to obtain the sparse loading values A involve selecting the best model $\hat{\delta_{k}}$ by choosing the values of $l_{1}$ and $l_{2}$ to achieve a simple model with a cumulative variance proportion of at least 80%. Therefore, in this study, $l_{1}$=0.009 and $l_{2}$=0.0001 were used. This is because both values have provided a cumulative variance proportion that has exceeded 80% which is 81.8%. Next, the resulting sparse loading values are as in Table 5.

**Table 5:** Optimal sparse loading value.

Loading value	${Z_{1}}^{*}$	${Z_{2}}^{*}$	${Z_{3}}^{*}$	${Z_{4}}^{*}$	${Z_{5}}^{*}$
${X_{1}}^{*}$	0.060	0	−0.670	0	0
${X_{2}}^{*}$	0.695	0	0	0	0
${X_{3}}^{*}$	0.650	0	0	0	0
${X_{4}}^{*}$	−0.046	0	−0.527	0	0
${X_{5}}^{*}$	−0.146	0	−0.419	0	0
${X_{6}}^{*}$	0	0	0	−0.561	0
${X_{7}}^{*}$	0	0	0	−0.607	0
${X_{8}}^{*}$	0	0	0	−0.500	0
${X_{9}}^{*}$	0	0	0	0	0.708
${X_{10}}^{*}$	0	0	0	0	0.665
${X_{11}}^{*}$	0	−0.538	0	0	0
${X_{12}}^{*}$	0	−0.568	0	0	0
${X_{13}}^{*}$	0	0.570	0	0	0
					

The obtained optimal sparse loading values are simpler because each $Z_{k}^{*}$ consists only of a few $X_{j}^{*}$ variables that are considered to contribute significantly to the formation of $Z_{k}^{*}$, while $X_{j}^{*}$ variables that are deemed not to contribute significantly are ignored, as indicated by the weights $A_{\mathrm{jk}}$ being zero. As shown in Table 5, there is also an effect of grouping $X_{j}^{*}$ variables with high correlation in each $Z_{k}^{*}$, meaning that the formation of the optimal sparse principal component $Z_{k}^{*}$ can identify groups of $X_{j}^{*}$ variables that have a similar impact on data variation.

After the optimal sparse principal components $Z_{k}^{*}$ are formed, we can obtain the sparse binary logistic regression estimates from categorical principal component analysis on the stunting risk event data, as shown in Table 6.

**Table 6:** Estimation results.

Variable	Coef. Estimation
Constant	−3.361
${Z_{1}}^{*}$	−2.137
${Z_{2}}^{*}$	0.791
${Z_{3}}^{*}$	0.484
${Z_{4}}^{*}$	1.014
${Z_{5}}^{*}$	−0.150

Based on Table 6, we can express the sparse categorical principal component logistic regression analysis model for the stunting risk event data as following Eq. (21).

(21a) $$g=-\;3.361-2.137{Z_{1}}^{*}+0.791{Z_{2}}^{*}+0.484{Z_{3}}^{*}+1.014{Z_{4}}^{*}-0.150{Z_{5}}^{*}$$

The feasibility test of the relationship model for the stunting risk factors, as shown in Eq. (21), was carried out using the likelihood ratio statistical test, namely $G^{2}$ at a significance level of *α* = 0.05. We obtained a value of $G^{2}=144.81\;$and $\chi^{2}=11.07$, which means that the sparse category principal component logistic regression model is worthy of explaining the relationship model of stunting risk factors with all the predictors studied. Furthermore, the level of accuracy of the model obtained based on the confusion matrix results in Table 7 is around 88.85%, and the sensitivity level is around 75.55%. This value is higher than the results of the categorical principal component logistic regression model without sparse, with an accuracy of around 78.71% and a sensitivity of around 57.77%. The sparse categorical principal component logistic regression model provides better results. more accurate compared to the non-sparse model.

**Table 7:** Confusion matrix.

Actual	Predicted with sparse		Predicted without sparse	
	1	0	1	0
1	34	11	26	19
0	22	229	44	207

These results indicate that the model can explain the relationship between the response and all the predictors studied. The next step is to transform Eq. (21) back into the X variables based on the relationship between each $Z_{k}^{*}$ and the corresponding categorical $X_{j}^{*}$. Table 5 shows that the response variable *Y* can be clearly expressed by the predictor variable *X*. The model describing the relationship between stunting events in Maginti District and the risk factors that may cause stunting events is shown in Eq. (22).

(22a) $$\begin{array}{ccc} g\; & = & -\;3.361-0.097X_{1.2}-0.404X_{2.2}-0.378X_{3.2}-0.022X_{4.2}+0.046X_{5.2}-0.149X_{6.2}\;-0.162X_{7.2}-0.133X_{8.2}-0.005X_{9.2}\\ & & -\;0.005X_{9.3}-0.004X_{10.2}-0.005X_{10.3\;}+0.101X_{11.2}-0.066X_{11.3}-0.036X_{11.4}+0.107X_{12.2}-0.069X_{12.3}\\ & & -\;0.037X_{12.4}-0.107X_{13.2})+0.070X_{13.3} \end{array}$$

Based on the estimated model for stunting risk factors obtained through sparse categorical principal component analysis logistic regression, several factors have more than a one-time higher risk of affecting stunting events. These factors include a history of illness, inadequate complementary feeding, formula milk consumption, and the lack of supplementary food and iron tablets for the mother. Regarding birth height and weight, we found that respondents with normal birth height have a 0.667 times lower risk of experiencing stunting compared to those with low birth height. Similarly, respondents with normal birth weight have a 0.685 times lower risk of experiencing stunting compared to those with low birth weight. Birth height and weight also play a role, with toddlers born with normal height and weight being at a lower risk of stunting than those with low birth height and weight. This highlights the importance of monitoring the health of both toddlers and mothers during pregnancy, mainly by providing additional nutrition and supplements for mothers.

The results of this study have provided knowledge related to several factors that have a major influence on the indication of stunting in toddlers through a statistical inferential approach. Child factors, such as birth weight and height, medical history, breast milk intake, and age of additional food provision, are the dominant factors causing more significant stunting. Previous studies have found that various diseases, such as diarrhea, respiratory tract infections, and fever, contribute to child growth [26]. However, maternal factors also significantly influence the growth and development of their children, including nutritional intake and iron tablets during pregnancy. For the case of stunting, this study can be further developed by adding data sets and variables that have not been studied, such as childcare factors, economy, environment, and other factors. The variety of factors that can influence the risk of stunting means that method development can be carried out in further studies, for example, mixed effects derived from predictors.

## Limitations

The method applies to categorical data in both the response and its predictors. In addition, a high correlation occurs between predictors, which is called multicollinearity.

## Ethics statements

This research involved human subjects, and in the data collection process, it has gone through Research Permit No. 11,043/UN4.11.7/PT.01.04/2023 from the Department of Statistics.

## Supplementary material *and/or* additional information [OPTIONAL]

None.

## CRediT authorship contribution statement

**Anna Islamiyati:** Conceptualization, Methodology, Formal analysis, Writing – original draft. **Muhammad Nur:** Formal analysis. **Abdul Salam:** Investigation. **Wan Zuki Azman Wan Muhamad:** Software. **Dwi Auliyah:** Data curation, Project administration.

## Declaration of competing interest

The authors declare that they have no known competing financial interests or personal relationships that could have appeared to influence the work reported in this paper.

## Data Availability

Data will be made available on request.
